# Effects of resisted sprint training on sprint, jump, and change-of-direction performance in athletes: a systematic review and meta-analysis

**DOI:** 10.3389/fphys.2025.1711992

**Published:** 2025-11-26

**Authors:** Chengcheng Li, Lunxin Chen, Qin Zhang

**Affiliations:** 1 School of Physical Education and Sports, Anhui Normal University, Wuhu, China; 2 School of Physical Education and Sports, Central China Normal University, Wuhan, China; 3 School of Physical Education, Anqing Normal University, Anqing, China

**Keywords:** resisted sprint training, unresisted sprint training, change-of-direction, vertical jump, sprint

## Abstract

**Objective:**

To systematically evaluate the effects of RST (resisted sprint training) on athletes’ sprint, jump, and COD (change-of-direction performance).

**Methods:**

Following PRISMA, we searched seven databases from inception to 12 July 2025—PubMed, Web of Science (all databases), MEDLINE, CENTRAL, SPORTDiscus, Scopus, and Embase. Study certainty was appraised with GRADE. All included data were analyzed in Stata/MP 18.0.

**Results:**

Sixteen studies (404 participants) were included. RST significantly improved linear sprint performance (SMD = 0.65, p < 0.001, I^2^ = 21.2%), vertical jump performance (SMD = −0.38, p = 0.013 I^2^ = 0.0%), and COD ability (SMD = 1.10, p < 0.001, I^2^ = 0.0%). UST (unresisted sprint training)also significantly improved linear sprint performance (SMD = 0.42, p < 0.001, I^2^ = 15.6%) and COD ability (SMD = 0.60, p < 0.001, I^2^ = 0.0%), but not vertical jump performance (SMD = −0.03, p = 0.872, I^2^ = 0.0%). Compared with UST, RST produced a greater improvement in COD ability (p = 0.043), with no significant differences for linear sprint (p = 0.057) or vertical jump (p = 0.102). Subgroup analyses indicated that, relative to UST, RST had larger benefits for 0–10 m linear sprint performance (p < 0.001) and among youth athletes (p = 0.000). Versus RT (regular training), RST yielded greater improvements in linear sprint performance (p < 0.001) and vertical jump performance (p < 0.001), but not in COD ability (p = 0.064).

**Conclusion:**

Both RST and UST improve linear sprint performance and COD ability. Only RST shows a significant within-group gain in vertical jump. Compared with UST, RST yields greater benefits for initial acceleration (0–10 m) and among youth athletes. Practically, when the primary goal is initial acceleration and COD, prioritize RST; for broader explosive-power development, pair RST with vertically oriented strength or plyometric training to develop both horizontal and vertical force qualities.

**Systematic Review Registration:**

https://www.crd.york.ac.uk/prospero/#guidancenotes, identifier PROSPERO (CRD420251103833).

## 1 Introduction

In both track and team sports, lower-limb explosive abilities—including sprinting speed, vertical jump capacity, and change-of-direction (COD)ability—are critical determinants of athletic success (Nygaard Falch, Guldteig Rædergård et al., 2019; Wheeler Botero, Patiño Palma et al., 2023). For instance, in sprint events ranging from 60 to 400 m, sprint velocity often determines the outcome (Bezodis et al., 2019), while in field-based sports such as soccer and rugby, rapid acceleration and efficient COD are essential for gaining a competitive edge (Nygaard Falch, Guldteig Rædergård et al., 2019). Likewise, vertical jump performance plays a decisive role in basketball rebounding and aerial duels in soccer, reflecting not only lower-limb power but also enhancing control over aerial balls (Wheeler Botero, Patiño Palma et al., 2023). Collectively, sprinting, jumping, and COD abilities represent an integrated manifestation of lower-limb neuromuscular power output, as evidenced by shared force–velocity–power determinants across sprint and jump tasks and their associations with COD performance (Loturco et al., 2015; Morin and Samozino, 2016; Jiménez-Reyes et al., 2017; Nimphius et al., 2018; Lin et al., 2023; Wheeler Botero, Patiño Palma et al., 2023).

In practice, unresisted sprint training (UST) and resisted sprint training (RST) are frequently employed to comprehensively enhance athletes’ explosive performance (Petrakos et al., 2016; Alcaraz et al., 2018; Myrvang and van den Tillaar, 2024). UST reflects the traditional sport-specific sprinting pattern and is typically performed without additional load (Zabaloy et al., 2025). By contrast, RST applies external resistance during sprinting—such as sled towing, weighted vests, or resistance parachutes—to intensify neuromuscular stimulation and develop sprint-specific force, particularly horizontal propulsive capacity (Petrakos et al., 2016).

Although RST is theoretically expected to enhance sprint performance, its superiority over UST remains uncertain. Early systematic reviews demonstrated that RST effectively improves sprinting, particularly during the initial acceleration phase, but did not establish superiority over UST (Alcaraz et al., 2018). In contrast, experimental evidence has shown that RST significantly reduces 10-m sprint times and provides a statistical advantage over UST in short-distance acceleration (Myrvang and van den Tillaar, 2024). This finding is reinforced by randomized and controlled trials in rugby and soccer that have reported larger pre–post gains at 10 m and 30 m with RST than with UST, with effects often more pronounced at 5–10 m under individualized heavy-load prescriptions (West et al., 2013; Derakhti et al., 2022; Panasci et al., 2023); recent meta-analytic evidence points in the same direction (Dougan et al., 2025). Overall, while current research supports the effectiveness of RST in improving sprint performance, consensus on whether it outperforms UST is lacking. In practical terms, current evidence indicates that benefits are most consistent in the acceleration domain, particularly at 0–10 m and, to a lesser extent, 10–30 m (Haugen et al., 2019), whereas differences versus unresisted sprinting become small or non-significant beyond 30 m as athletes approach near-maximal velocity (Alcaraz et al., 2018; Myrvang and van den Tillaar, 2024).

Beyond linear sprint speed, sport context also moderates RST’s effects. In team sports (e.g., soccer, rugby), trials show consistent RST benefits for acceleration and COD, with very-heavy sled loading favoring COD in youth soccer (Baena-Raya et al., 2025). Among track sprinters, RST typically reduces 10 m time but adds little at 20 m or near-maximal velocity versus unresisted or assisted sprinting (Myrvang and van den Tillaar, 2024). For jump-dominant sports (e.g., basketball, volleyball), vertical-jump responses are inconsistent (Prieske et al., 2018). Scholars have examined the broader impact of RST on lower-limb explosive performance. For instance, an 8-week RST program—particularly under heavy-load conditions—was found to significantly improve 505 change-of-direction test performance in youth soccer players (Baena-Raya et al., 2025). However, evidence for vertical-jump adaptations with RST is mixed: several randomized or controlled trials report no between-group advantage for RST over unresisted sprinting (Rey et al., 2017; Prieske et al., 2018; Pareja-Blanco et al., 2020). Taken together, these findings suggest that RST adaptations may be direction-specific, primarily enhancing horizontal actions such as acceleration and COD, while offering more limited transfer to vertical performance (Junge et al., 2023; Panasci et al., 2023). Given the inconsistencies across studies, further high-quality trials in diverse athlete populations are needed to clarify the multidimensional effects of RST and its underlying mechanisms.

In summary, although current evidence indicates that resisted sprint training (RST) can effectively improve sprint acceleration, its superiority over UST has not been consistently confirmed. Moreover, the benefits of RST for vertical jump performance and COD ability require further validation. Building on this gap, the present study systematically compared the effects of RST and UST, evaluating their impact on sprint acceleration, vertical jump performance, and COD ability. Subgroup analyses further examined differences across sprint distances and age groups. These findings aim to provide coaches with evidence-based guidance for designing more targeted sprint and explosive performance training programs.

## 2 Methods

### 2.1 Protocol and registration

The protocol was prepared in line with PRISMA-P and prospectively registered with PROSPERO (CRD420251103833).

### 2.2 Eligibility criteria

The inclusion and exclusion criteria for this study were predefined according to the PICOS framework (Participants, Interventions, Comparisons, Outcomes, and Study design). They were developed in line with the PRISMA 2020 statement and the Cochrane Handbook for Systematic Reviews of Interventions to ensure methodological rigor, consistency, and transparency in the selection of eligible studies (Table 1).

**TABLE 1:** Inclusion and exclusion criteria.

Category	Inclusion criteria	Exclusion criteria
Population	Competitive or recreational athletes	Non-athletes or individuals with functional limitations
Intervention	Studies involving resisted sprint training (RST), including sled towing, parachute sprinting, uphill sprinting, weighted vest, or tethered sprint protocols RST is defined as sprinting under externally applied resistance to enhance neuromuscular output and sprint-specific force production (Petrakos et al., 2016; Myrvang and van den Tillaar, 2024)	Interventions not clearly identifiable as RST, or involving multiple simultaneous methods that confound the effects of RST.
Comparator	Comparator groups included unresisted sprinting, conventional training. The control group unresisted sprinting performed only sport-specific training	Lack of proper comparator group or use of confounding interventions
Outcome	Studies reporting any sprint-related performance outcomes, including jump ability, sprint time, acceleration, or change-of-direction ability	Studies not reporting quantitative data (e.g., no mean ± SD), or no sport performance indicators
Study design	Randomized and non-randomized controlled trials included	Reviews, conference abstracts, patent, protocol,case reports, or animal studies excluded

### 2.3 Information sources

This study was conducted in accordance with the PRISMA 2020 statement and the Cochrane Handbook for Systematic Reviews of Interventions. A comprehensive search was performed across major medical and sport science databases. The search strategy was informed by established practices in previous systematic reviews and constructed using relevant subject terms combined with Boolean operators. The following databases were searched: Web of Science (All Databases), PubMed, Embase, MEDLINE, CENTRAL (Cochrane Central Register of Controlled Trials), Scopus, and SPORTDiscus. The search period covered the inception of each database through 12 July 2025.

### 2.4 Search strategy

A structured search strategy was developed across multiple databases, using Boolean operators (AND, OR) in combination with keywords and MeSH terms. The strategy was informed by existing systematic reviews and iteratively refined to align with the study objectives. The main keywords included: “resisted sprint training,” “resisted sprinting,” “sled sprint,” “parachute sprint,” “tethered sprint,” “uphill sprint,” “1,080 sprint,” “sprint performance,” “acceleration,” “speed development,” “sprint time,” and “sports performance.” Keyword combinations employed both free-text terms and controlled vocabulary, with database-specific adaptations as needed. For illustration, the PubMed search strategy is provided in Table 2. The complete search strategies for all databases are included in the Supplementary Material S1.

**TABLE 2:** Search strategy examples.

#1	“resisted sprint” (Title/Abstract) OR ((“resist” (All fields) OR “resistance” (All fields) OR “resistances” (All fields) OR “resistant” (All fields) OR “resistants” (All fields) OR “resisted” (All fields) OR “resistence” (All fields) OR “resistences” (All fields) OR “resistent” (All fields) OR “resistibility” (All fields) OR “resisting” (All fields) OR “resistive” (All fields) OR “resistively” (All fields) OR “resistivities” (All fields) OR “resistivity” (All fields) OR “resists” (All fields)) AND “running” (Title/Abstract)) OR “sled train*” (Title/Abstract) OR “sled tow*” (Title/Abstract) OR “sled pull*” (Title/Abstract) OR “weighted sled” (Title/Abstract) OR ((“aviation” (MeSH Terms) OR “aviation” (All fields) OR “parachuting” (All fields) OR “parachute” (All fields) OR “parachuted” (All fields) OR “parachutes” (All fields)) AND “sprint*” (Title/Abstract)) OR “weighted vest” (Title/Abstract) OR “tethered sprint” (Title/Abstract) OR ((“harness” (All fields) OR “harnessed” (All fields) OR “harnesses” (All fields) OR “harnessing” (All fields)) AND “sprint” (Title/Abstract)) OR “uphill sprint*” (Title/Abstract) OR ((“inclinable” (All fields) OR “inclination” (All fields) OR “inclinations” (All fields) OR “incline” (All fields) OR “inclined” (All fields) OR “inclines” (All fields) OR “inclining” (All fields)) AND “sprint*” (Title/Abstract))
#2	“jump” (All Fields) OR “vertical jump” (All Fields) OR “jump height” (All Fields) OR “countermovement jump” (All Fields) OR “CMJ” (All Fields) OR “agility” (All Fields) OR “change-of-direction” (All Fields) OR “change-of-direction” (All Fields) OR “COD” (All Fields) OR “505 test” (All Fields) OR “T-test” (All Fields) OR “sprint performance” (All Fields) OR “sprint speed” (All Fields) OR “acceleration” (All Fields)
#3	“athlete*” (All Fields) OR “player*” (All Fields) OR “team sport*” (All Fields) OR “sportspeople” (All Fields)
#4	#3 AND #2 AND #1

### 2.5 Selection process

All retrieved records were imported into EndNote X9 (Clarivate Analytics) for automatic duplicate removal. Titles and abstracts were then screened to exclude studies that were clearly irrelevant. To ensure accuracy, full texts of potentially eligible studies were reviewed, and corresponding authors were contacted when additional details were required. The final list of included studies was exported to Microsoft Excel for data extraction and organization. Study selection was conducted independently by two reviewers (L.C. and C.L.). Any disagreements were resolved through adjudication and verification by a third reviewer (Z.Q.).

### 2.6 Data collection process

Following the predefined study protocol, we developed a standardized electronic data extraction form. Prior to full-scale extraction, the form was piloted on three included studies to evaluate its clarity and completeness, and subsequently refined as needed. Data extraction was performed collaboratively by three reviewers. Two researchers (L.C. and C.L.) independently organized all eligible studies in a structured Microsoft Excel sheet and extracted the following information: (1) study characteristics (first author, year of publication, country); (2) participant details (sample size, age, intervention period); (3) intervention features (experimental and control group protocols, duration, frequency, and training cycle); (4) outcomes (e.g., performance measures); and (5) information relevant to risk of bias assessment (e.g., randomization, blinding, completeness of data). A third reviewer (C.L.) checked and verified all extracted information to ensure accuracy. Any discrepancies were adjudicated by a senior reviewer (Z.Q.), who confirmed the final dataset. For studies presenting results solely in graphical format, data were extracted using GetData Graph Digitizer software (Osthold Software, Kiel, Germany). When critical data (e.g., means or standard deviations) were unclear, missing, or unreported, we contacted the corresponding authors by email (at least twice, 2 weeks apart). If no response was received, ResearchGate was used as an alternative platform. Studies for which essential data could not be obtained prior to manuscript submission were excluded.

### 2.7 Data items

The predefined outcomes extracted in this review were linear sprint performance, vertical jump ability, and change-of-direction (COD) performance. The specific indicators extracted from each included study are summarized in Table 3.

**TABLE 3:** Extracted indicators from included studies.

Outcomes categories	Measure
Linear Sprint Performance	0–30 m linear sprint time (5m、10m、15m、20m、30 m)
Vertical Jump Performance	Countermovement Jump (CMJ),Squat Jump (SJ)
change-of-direction (COD) Performance	505 change-of-direction Test (T505), change-of-direction Time (COD time),T-Half Test

### 2.8 Study risk of bias assessment

In this systematic review, the risk of bias for all included studies was assessed using the Cochrane Risk of Bias tool (version 2.0) (Sterne et al., 2019). This tool evaluates potential sources of bias across several key domains: the randomization process, allocation concealment, blinding, completeness of outcome data, and selective outcome reporting. Each study was assigned an overall risk of bias rating in one of three categories: *low risk* (all domains rated as low risk), *high risk* (at least one domain rated as high risk), or *some concerns* (studies that did not meet the criteria for either of the other two categories). Two reviewers (L.C. and C.Y.) independently conducted the assessments. Any disagreements were resolved through discussion, and if consensus could not be reached, a third reviewer (Z.Q.) adjudicated and confirmed the final judgment.

### 2.9 Effect measures

Following the *Cochrane Handbook for Systematic Reviews of Interventions* (version 6.3, section 6.5.1), the standardized mean difference (SMD) was selected as the summary effect size when different measurement tools were used across trials to assess the same outcome (Higgins et al., 2023). Therefore, the standardized mean difference (SMD) was used as the summary effect size. The interpretation thresholds for SMD were as follows: trivial (SMD = 0.20), small (0.20 ≤ SMD = 0.50), moderate (0.50 ≤ SMD = 0.80), and large (SMD ≥0.80) (Hedges and Olkin, 2014). All pooled effect sizes were reported with their 95% confidence intervals, and statistical significance was defined as p = 0.05 (Higgins et al., 2023).

### 2.10 Synthesis methods

The pooled results were presented visually using forest plots. All meta-analyses were conducted with a random-effects model, reflecting the anticipated clinical and methodological heterogeneity across studies, including differences in participant characteristics, intervention protocols, and outcome measurements. Although the *Cochrane Handbook for Systematic Reviews of Interventions* does not prescribe a universal recommendation for model selection, we opted for a random-effects approach to minimize the risk of underestimating heterogeneity that may occur with a fixed-effects model. Statistical heterogeneity was assessed using the I^2^ statistic. Values of I^2^ below 25% were considered negligible, those between 25% and 75% indicated moderate heterogeneity, and values above 75% reflected substantial heterogeneity. A p-value = 0.05 was regarded as evidence of statistically significant heterogeneity (Higgins et al., 2003; Borenstein, 2022).

Three predefined subgroup analyses were conducted to examine potential sources of heterogeneity: (i) comparison type (RST vs. UST; RST vs. RT); (ii) sprint distance (0–10 m, 0–20 m, 0–30 m); and (iii) participant age (youth athletes vs. adults). Subgroup pooling was only performed when at least two studies were available. Publication bias was assessed following the Cochrane Handbook, which recommends that robust evaluation of small-study effects requires ≥10 studies. Accordingly, funnel plots and Egger’s regression test were performed only when at least ten studies were available. To test the robustness of the findings, sensitivity analyses were performed for all primary outcomes. However, the small number of studies in certain subgroups—two in the vertical jump–RT group, three in the COD–UST group, and two in the COD–RT group—limits the interpretability of these subgroup results. All statistical analyses were conducted using Stata/MP 18.0 (StataCorp LLC, College Station, TX, United States).

### 2.11 Reporting bias assessment

For outcomes with ten or more included studies, publication bias was evaluated using funnel plots combined with Egger’s regression test for asymmetry. Funnel plots were visually inspected to assess symmetry, and Egger’s test was applied to provide quantitative verification, with p = 0.05 indicating significant asymmetry. For outcomes with fewer than ten studies, formal statistical tests for publication bias were not conducted, in line with the recommendations of the *Cochrane Handbook for Systematic Reviews*, to avoid the risk of misleading results due to insufficient test power.

### 2.12 Certainty assessment

Two reviewers (L.C. and C.Y.) independently assessed the quality of evidence using the GRADEpro-GDT online platform (www.gradepro.org). Following the GRADE framework, the quality of evidence for each outcome was categorized as high, moderate, low, or very low, based on the overall confidence in the effect estimates. By default, randomized controlled trials were considered high-quality evidence, but could be downgraded in five domains: risk of bias, inconsistency, indirectness, imprecision, and publication bias. Any disagreements between the two reviewers were first resolved through discussion; if consensus could not be reached, a third reviewer (Z.Q.) acted as arbiter.

## 3 Results

### 3.1 Study selection

**FIGURE 1:**
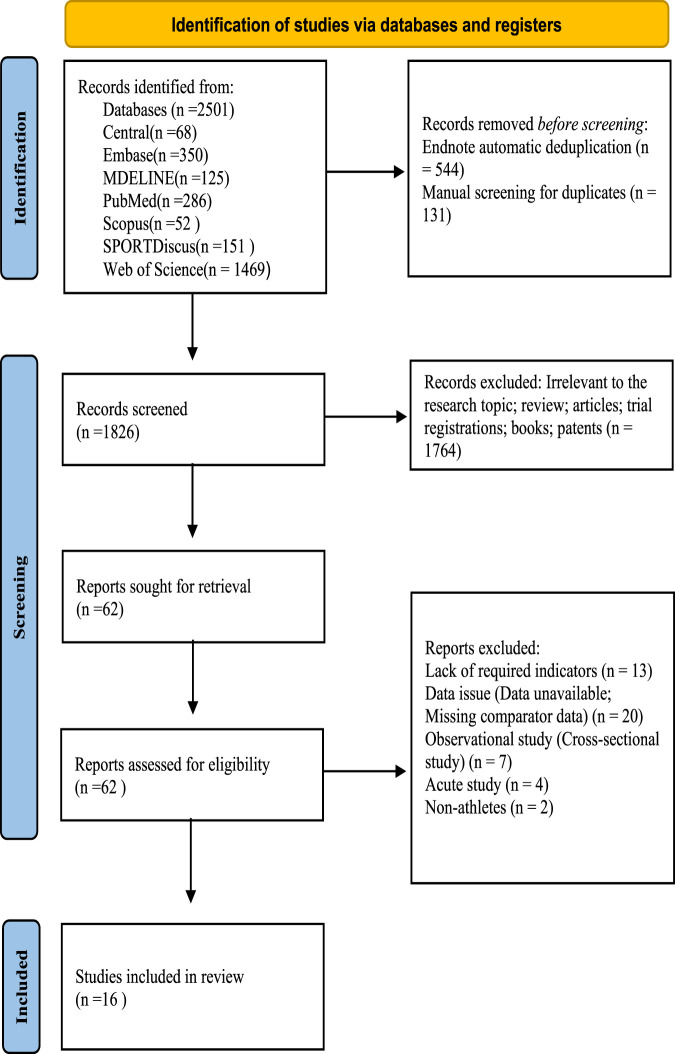
Schematic representation of the literature selection and review protocol.

A total of 2,501 records were initially identified through database searches. After removing 544 duplicates automatically using EndNote software and an additional 131 duplicates by manual screening, 1,826 unique records remained. Following title and abstract screening, 1,764 studies were excluded for reasons such as irrelevance to the topic, being reviews, trial registrations, books, or patents. The remaining 62 records were retrieved in full text for eligibility assessment. Of these, 46 were excluded for the following reasons: lack of required outcomes (n = 13), insufficient or unavailable data (n = 20), cross-sectional design (n = 7), pilot studies (n = 4), or non-athlete populations (n = 2). Ultimately, 16 studies met the inclusion criteria and were included in both the systematic review and meta-analysis. The detailed study selection process is illustrated in Figure 1.

### 3.2 Study characteristics

This review included 16 studies with a total of 404 participants, comparing the effects of RST with UST and regular training (RT) on lower-limb speed and power in athletes. Participants were predominantly competitive athletes from various sports, representing both youth and adult groups, and included both male and female subjects. RST interventions varied and included sled towing, parachute resistance, elastic bands, and motorized resistance devices. The control groups consisted of UST (sprinting without external load) or RT (technical, tactical, or general conditioning training without sprint-specific resistance). Training interventions lasted 4–12 weeks, with a frequency of 2–4 sessions per week. Detailed characteristics of the included studies are presented in Table 4.

**TABLE 4:** Study characteristics and intervention details.

Study	Sport/Population	Age group	Intervention	Load/Intensity	Frequency	Duration	Control	Outcome Type
Aloui et al. (2019)	Handball players	Adult (male)	ERST	Elongation 200%/250%	2/week	8 weeks	RT	0–5 m 0–30 m T-half test CMJ Height SJ Height
Baena-Raya et al. (2025)	Soccer players	Youth (male)	SRST	Vec25%/75%	2/week	8 weeks	RT	0–10 m 0–20 m 0–30 m 10–20 m 20–30 m
Carlos-Vivas et al. (2020)	Soccer players	Youth (male)	WVST/MRST/CRST	10–20%BW	2/week	8 weeks	UST	0–10 m 10–20 m 20–30 m 0–30 m
Lahti et al. (2020)	Soccer players	Adult (male)	SRST	Vec60%/50%	1–2/week	11 weeks	RT	0–5 m 0–10 m 0–20 m 0–30 m
Le Scouarnec et al. (2022)	Soccer players	Youth (male)	ERST	—	1–2/week	10 weeks	UST	0–5 m 0–10 m 0–20 m 0–30 m 20–30 m
Luteberget et al. (2015)	Handball players	Adult (Female)	SRST	12.4% ± 0.2% BW	2/week	10 weeks	UST	0–10 m 0–30 m
Makni et al. (2024)	Handball players	Youth (male)	WVST/ALWT	12.6%BW/load1.44 kg	3/week	6 weeks	UST	0–10 m 0–30 m T-half test SJ Height CMJ Height
Martinopoulou et al. (2011)	Sprinters	Adult (male)	PST	—	3/week	4 weeks	UST	0–10 m 10–20 m 0–20 m
Morin et al. (2017)	Soccer players	Adult (male)	SRST	80%BW	2/week	8 weeks	UST	0–5 m 0–20 m
Moya-Ramon et al. (2020)	Tennis players	Youth (male)	CRST	10–15%BW	2/week	6 weeks	UST	0–20 m T505ND T505D CMJ Height
Panasci et al. (2023)	Rugby players	Adult (male)	SRST	12.6%BW	2/week	8weeks	UST	0–10 m 0–30 m
Rey et al. (2017)	Soccer players	Adult (male)	WVST	18.9% ± 2.1%BW	2/week	6 weeks	UST	0–10 m 0–30 m CMJ Height
Spinks et al. (2007)	Systematically trained athletes	Adult (male)	SRST/UST	Vec10%	2/week	8weeks	UST/RT	0–5 m 5–10 m 10–15 m 0–15 m CMJ Height
Stavridis et al. (2023)	Sprinters	Adult (male + Female)	SRST	Vec50%/10%BW	2/week	6 weeks	UST	0–5 m 0–10 m 0–15 m 0–20 m 0–30 m
West et al. (2013)	Rugby players	Adult (male)	SRST	12.6%BW	2/week	6 weeks	UST	0–10 m 0–30 m
Zisi et al. (2022)	Sprinters	Youth (male + Female)	SRST	Vec50%	—	Four experimental days	RT	0–5 m 5–10 m 10–15 m

### 3.3 Risk of bias, certainty of evidence

**FIGURE 2:**
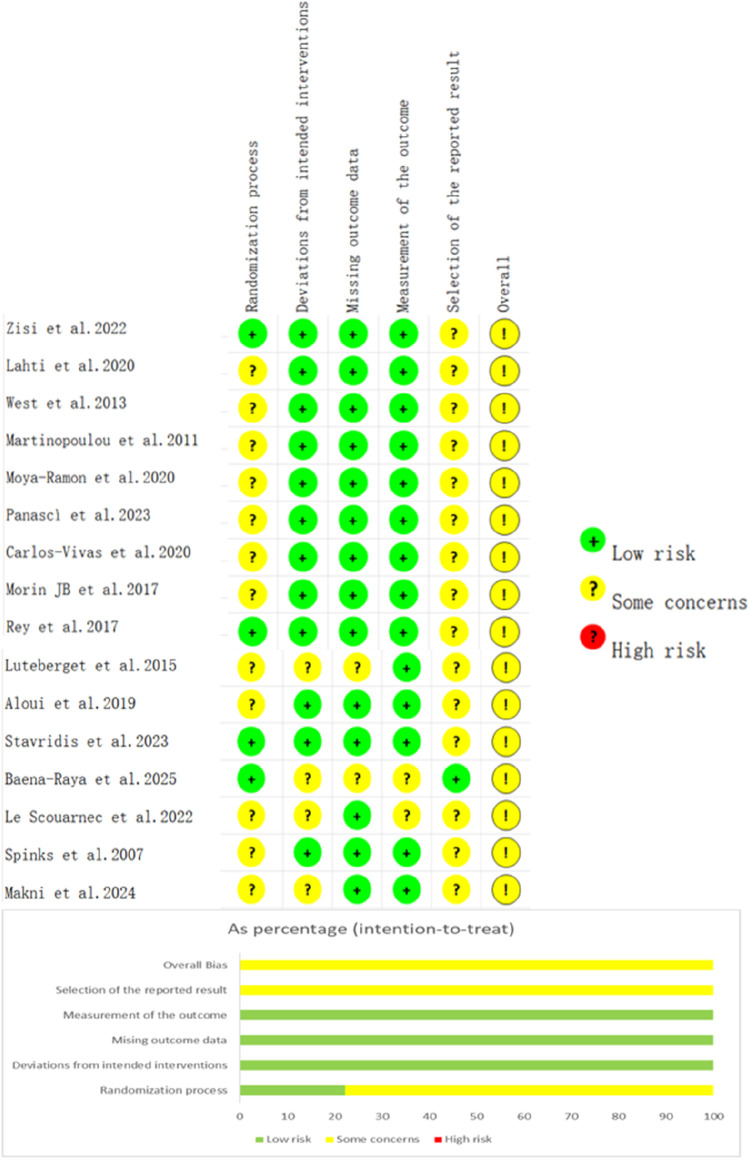
Overall risk of bias across studies (% distribution).

The risk of bias assessment is shown in Figure 2. Overall, all included studies were rated as presenting “some concerns.” For the randomization process, most studies were judged as having “some concerns,” primarily due to insufficient reporting of allocation concealment and baseline comparability. Only a few studies were rated as “low risk” because they provided clear descriptions of sequence generation and allocation procedures. With respect to deviations from intended interventions, most studies were rated as “low risk,” as no systematic deviations attributable to awareness or execution issues were identified. For missing outcome data, the overall risk was “low,” although two studies were rated as having “some concerns” because they did not clearly report dropout numbers or reasons (Le Scouarnec et al., 2022; Baena-Raya et al., 2025). Regarding outcome measurement, most studies were rated as “low risk,” given that outcome indicators were objective and measurement procedures were standardized, with no evidence of systematic bias from measurement methods. However, the risk of bias due to selective reporting was more prevalent, mainly because of the absence of pre-registration or insufficient justification for outcome selection, which raised the possibility of selective reporting.

According to the GRADE framework, the certainty of evidence for all prespecified outcomes was assessed using the GRADEpro-GDT platform (Table 5). For sprint performance (16 studies), the certainty of evidence was rated as high. The overall risk of bias was low, heterogeneity across studies was minimal, the participants and interventions demonstrated good directness, effect estimates were precise, and no evidence of publication bias was detected; therefore, no downgrading was applied. For vertical jump performance (6 studies), the certainty of evidence was rated as moderate, downgraded by one level due to inconsistency. While there were no major issues related to risk of bias, indirectness, imprecision, or publication bias, the presence of moderate statistical heterogeneity reduced confidence in the pooled estimate. For COD ability (5 studies), the certainty of evidence was also rated as moderate, downgraded for inconsistency. The overall risk of bias was low, the study designs and interventions demonstrated good directness, and the effect estimates were relatively precise without clear evidence of publication bias. However, variability in study results reduced the overall certainty of the evidence.

**TABLE 5:** GRADE summary of evidence.

Outcome	Number of studies	Risk of bias	Inconsistency	Indirectness	Imprecision	Publication bias	Overall certainty (GRADE)
Linear sprinting performance	16	Not serious	Not serious	Not serious	Not serious	Not suspected	⨁⨁⨁⨁ High
Vertical jump performance	6	Not serious	serious	Not serious	Not serious	Not suspected	⨁⨁⨁ Moderate
Change-of-direction ability	5	Not serious	serious	Not serious	Not serious	Not suspected	⨁⨁⨁ Moderate

## 4 Meta-analysis result

### 4.1 Linear sprint performance

**FIGURE 3:**
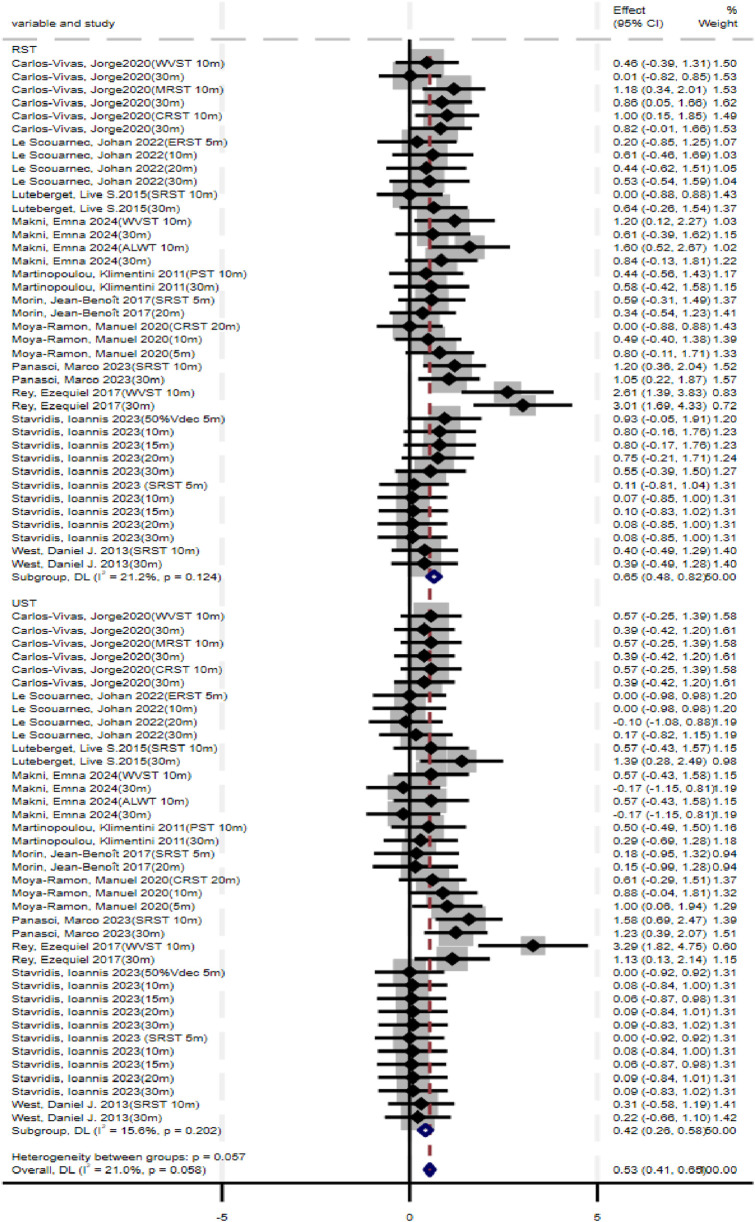
Forest plot of linear sprint performance (RST vs. UST).

Meta-analysis results (Figure 3) showed that both RST (SMD = 0.65, 95% CI: 0.48–0.82, p < 0.001) and UST (SMD = 0.42, 95% CI: 0.26–0.58, p < 0.001) significantly improved athletes’ linear sprint performance. No significant heterogeneity was observed for either RST (I^2^ = 21.2%, p = 0.124) or UST (I^2^ = 15.6%, p = 0.202). Compared with UST, the difference in improvement between the two interventions was not statistically significant (p = 0.057).

In addition, subgroup analyses (Table 6) indicated that, relative to UST, RST produced significantly greater improvements in 0–10 m sprint performance (p < 0.001) and among youth athletes (p < 0.001). Compared with RT, RST also showed significantly greater benefits for linear sprint performance (p < 0.001).

**TABLE 6:** Subgroup analysis result.

Variable	Data size	SMD	95%Cl	P_(Subgroup)_	P_(Interaction)_
Training type (by control)					
RST	17	1.03	0.80,1.27	0.000	0.000
RT	17	0.18	0.00,0.36	0.047	
Age group					
Youth athlete	17	0.81	0.63,1.00	0.000	0.000
Adult athlete	22	0.72	0.52,0.92	0.000	
Distance-specific sprint performance					
0–10m	14	0.81	0.49,1.13	0.000	0.000
0–20m	6	0.34	−0.04,0.73	0.080	
0–30m	12	0.71	0.37,1.05	0.000	

### 4.2 Vertical jump performance

**FIGURE 4:**
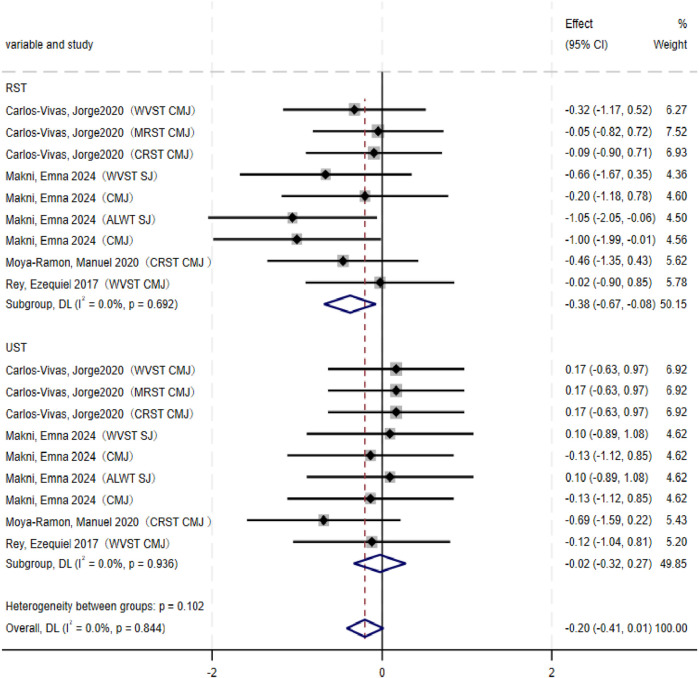
Forest plot of vertical jump performance (RST vs. UST).

Meta-analysis results (Figure 4) showed that RST significantly improved athletes’ vertical jump performance (SMD = −0.38, 95% CI: −0.67∼-0.08, p = 0.013). In contrast, UST did not significantly improve vertical jump ability (SMD = −0.03, 95% CI: −0.32–0.27, p = 0.872). No significant heterogeneity was observed for either RST (I^2^ = 0.0%, p = 0.692) or UST (I^2^ = 0.0%, p = 0.936). When directly compared with UST, the effect of RST on vertical jump improvement was not statistically significant (p = 0.102).

However, subgroup analysis (Table 7) indicated that, relative to RT, RST produced a significantly greater improvement in vertical jump performance (p < 0.001).

**TABLE 7:** Subgroup analysis result.

Variable	Data size	SMD	95%CI	P_(Subgroup)_	P_(Interaction)_
Training type (by control)					
RST	4	−0.85	−1.26, −0.44	0.000	0.000
RT	4	−0.53	−0.92, −0.13	0.010	

### 4.3 Change-of-direction performance

**FIGURE 5:**
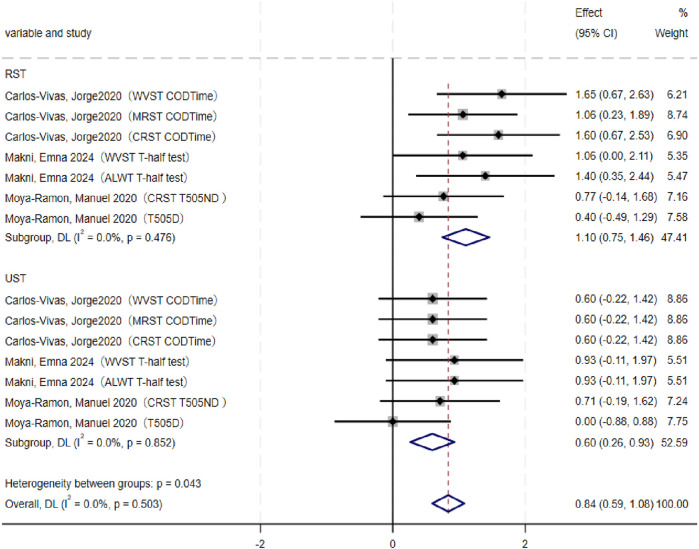
Forest plot of change-of-direction ability (RST vs. UST).

Meta-analysis results (Figure 5) indicated that both RST (SMD = 1.10, 95% CI: 0.75–1.46, p < 0.001) and UST (SMD = 0.*6*0, 95% CI: 0.26–0.93, p < 0.001) significantly improved athletes’ COD performance. No significant heterogeneity was observed for either RST (I^2^ = 0.0%, p = 0.476) or UST (I^2^ = 0.0%, p = 0.852). Compared with UST, RST produced a significantly greater improvement in COD ability (p = 0.043).

Subgroup analysis (Table 8) further showed that, relative to RT, the effect of RST on COD performance was not statistically significant (p = 0.064).

**TABLE 8:** Subgroup analysis result.

Variable	Data size	SMD	95%CI	P _(Subgroup)_	P_(Interaction)_
Training type (by control)					
RST	5	1.09	0.45, 1.73	0.000	0.000
RT	5	−0.53	−0.08, −0.47	0.686	

### 4.4 Publication bias

**FIGURE 6:**
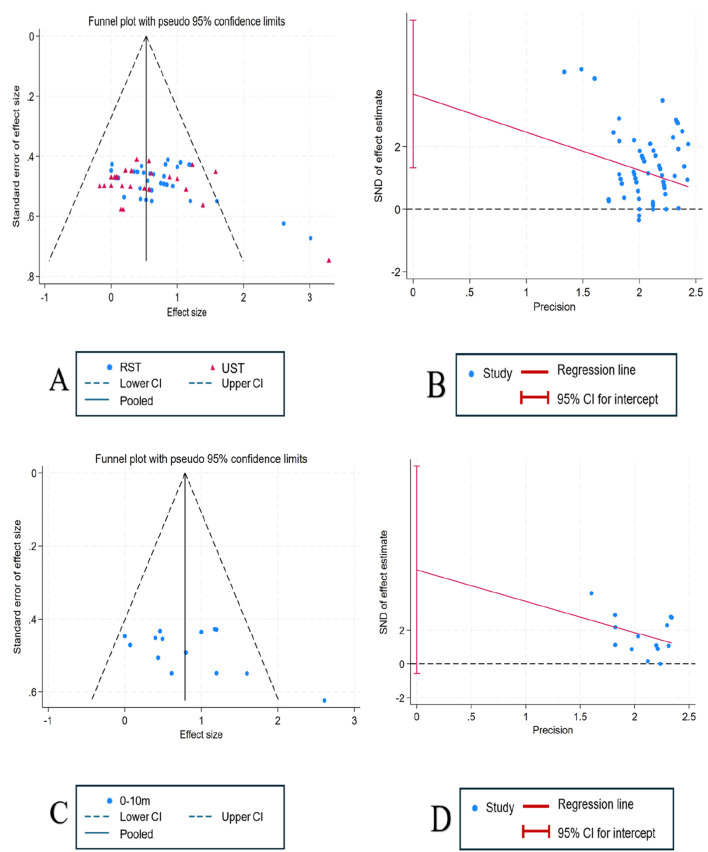
Evaluation of potential publication bias: funnel plot analysis and Egger’s test. **(A)** Funnel plot: linear sprint (RST vs. UST); **(B)** Egger’s test: linear sprint (RST vs. UST); **(C)** Funnel plot: 0–10 m sprint (RST vs. UST); **(D)** Egger’s test: 0–10 m sprint (RST vs. UST).

As shown in Figure 6, visual inspection of the funnel plot suggested possible publication bias. To further evaluate this, Egger’s regression test was performed. The results indicated potential publication bias for sprint performance (RST vs. UST) (t = 3.11, p = 0.003). However, after applying the trim-and-fill method, no additional studies were imputed (imputed = 0), and the pooled effect size remained unchanged (SMD = 0.55, 95% CI: −0.12–1.22), confirming the robustness of the findings. For the 0–10 m sprint performance, Egger’s test showed no significant evidence of publication bias (t = 1.98, p = 0.071).

## 5 Discussion

This systematic review and meta-analysis aimed to quantify and compare the effects of RST and UST on athletes’ linear sprint performance, vertical jump ability, and COD performance, and to further examine differences across sprint distances and athlete groups. The meta-analysis showed that RST significantly enhanced linear sprint performance (SMD = 0.65, p < 0.001), vertical jump ability (SMD = −0.38, p = 0.013), and COD performance (SMD = 1.10, p < 0.001). In contrast, UST did not significantly improve vertical jump ability (SMD = −0.03, p = 0.872), while producing significant gains in linear sprint performance (SMD = 0.42, p = 0.000) and COD performance (SMD = 0.60, p = 0.000).

Both RST and UST significantly enhanced linear sprint performance in our meta-analysis (RST: SMD = 0.65, p < 0.001; UST: SMD = 0.42, p < 0.001). Ward et al. (2024) likewise observed within-group RST improvements in early acceleration (SMD = −0.80) and late acceleration (SMD = −0.28) (Ward et al., 2024), while detecting no between-group difference versus unresisted sprinting across acceleration and near-maximal-velocity phases (note: negative SMD reflects faster times). Additionally, the pooled effect of RST on acceleration was (Hedges’ d ≈ 0.11, p = 0.08), indicating a small effect and no significant difference compared with an equivalent volume of unresisted sprint training (Aldrich et al., 2024). In the present analysis, when RT served as the control, RST (SMD = 0.53, p < 0.001) also produced significant improvements in sprint performance, further confirming its effectiveness. However, compared with UST, RST did not show a statistically significant advantage in overall sprint performance (p = 0.057), aligning with previous conclusions (Ward et al., 2024). Notably, RST demonstrated markedly greater benefits in 0–10 m sprint performance (SMD = 0.81vs.0.34vs.0.71, p < 0.001) and among youth athletes (SMD = 0.81vs.0.72, p < 0.001), with larger effect sizes. This suggests that RST is particularly effective in start acceleration and short-distance sprinting, whereas improvements in mid-phase sprinting appear less consistent. A likely explanation is that resisted sprinting shortens start time and enhances short-distance acceleration by specifically reinforcing horizontal force production and lower-limb drive (Alcaraz et al., 2018). Supporting this, another study reported that RST offers some advantage at the 10 m start phase but shows no significant differences at longer distances or peak sprinting speeds (Myrvang and van den Tillaar, 2024). Furthermore, adolescents show greater neuromuscular plasticity than adults (Granacher et al., 2016; Lesinski et al., 2016; Sánchez Pastor et al., 2023). In early–mid puberty, training gains are driven mainly by neural adaptations, with hypertrophy contributing more later (Granacher et al., 2016; Lesinski et al., 2016). Tendon properties are also more modifiable, as strength often rises faster than tendon stiffness (Mersmann et al., 2017). When loads are set so athletes run about 10%–20% slower than their normal unresisted speed, adolescents tend to respond particularly well. (Monte et al., 2017; Cahill et al., 2019; Osterwald et al., 2021). which likely explains the larger improvements observed in the adolescent subgroup. Based on this evidence, coaches are advised to prioritize RST in training programs targeting start acceleration and short-distance sprints, particularly for youth athletes, to leverage their greater neuromuscular adaptability.

This study found that UST did not significantly improve athletes’ vertical jump performance, whereas RST produced a significant enhancement, consistent with earlier findings (Junge et al., 2023). However, Amore and colleagues reported no significant improvements in soccer players following RST, which differs from our results (Amore et al., 2024). This discrepancy likely reflects how force is applied: early sprint acceleration benefits from the ability to push backward against the ground to move forward, whereas vertical jump performance leans more on upward force and the quality of the stretch–shortening cycle (SSC). Resisted-sprint work tends to sharpen that backward push during the first steps—so 0–10 m gains are common—but it may not strongly challenge SSC qualities, which can limit transfer to jump height (Morin et al., 2011; Nagahara et al., 2018; Samozino et al., 2022; Myrvang and van den Tillaar, 2024). By contrast, SSC-oriented plyometrics enhance vertical-jump performance via elastic energy storage and neuromuscular potentiation (Komi, 2000; Markovic, 2007; Cormie et al., 2011). Consistent with this, our analysis did not show a statistically significant advantage of RST over UST for vertical jump (p = 0.102), a pattern also seen in recent randomized controlled trials (Zabaloy et al., 2025). From a practical standpoint, combining light-load sled sprints with squats or jump squats, and integrating them with sprint and COD training, may provide complementary stimuli for both vertical and horizontal qualities. Under well-structured prescriptions, horizontally oriented resisted sprinting can also carry over to vertical power development (Bachero-Mena and González-Badillo, 2014; Pareja-Blanco et al., 2021).

Additionally, we observed that both RST and UST significantly improved athletes’ COD performance, consistent with previous findings (Gil et al., 2018). Compared with UST, RST produced larger improvements across various COD tests (SMD = 1.10vs.0.60, p = 0.043), leading to further reductions in COD time. This aligns with prior evidence (Hermosilla-Palma et al., 2025). Mechanistically, the re-acceleration demands of COD are similar to those of linear sprinting: following deceleration and turning, athletes must generate substantial horizontal force within a very short timeframe to re-accelerate (Morin et al., 2012; Dosʼ;Santos et al., 2017; McBurnie et al., 2022). By enhancing horizontal force output and start acceleration capacity, RST can effectively improve post-turn re-acceleration, thereby reducing COD time. This supports the perspective that linear acceleration is an important influencing factor of COD ability (Brughelli et al., 2008). Our meta-analysis corroborates this link, showing that gains in COD performance are closely tied to improvements in sprint acceleration. However, it should be noted that RST is inherently a linear sprint method, whereas COD requires multidirectional skills and high-intensity eccentric braking. Although RST demonstrates transfer effects to COD, its specificity remains under discussion. When control groups include sprint and agility drills, RST may not consistently show significant group-level advantages—a phenomenon reported in soccer and tennis athletes (Gil et al., 2018; Moya-Ramon et al., 2020). Therefore, these findings should be interpreted cautiously. From a practical standpoint, integrating RST with COD-specific drills and eccentric strength training is recommended (Forster et al., 2022).

We conducted sensitivity analyses on the meta-analysis results (Supplementary Material S3). For sprint performance, across the overall ≤30 m segments, including 0–10 m, 0–20 m, and 0–30 m, as well as youth and adult subgroups, stepwise exclusion of individual studies did not materially alter the pooled effect size. The combined estimates consistently fell within the 95% confidence intervals, supporting the robustness of the findings. For vertical jump, sensitivity analyses showed that the pooled effect of RST remained stable across all exclusion tests. Although the magnitude of effect fluctuated slightly, the overall direction and statistical significance were unchanged, reinforcing the reliability of the results. For change-of-direction ability, the pooled advantage of RST over UST remained stable under sensitivity analyses. Even after excluding any single study, the effect size retained statistical significance. This suggests that despite the limited number of included studies, the conclusion is consistent and robust. In summary, the sensitivity analyses confirmed that the main conclusions of this meta-analysis are robust and not dependent on any single study, thereby strengthening confidence in the available evidence.

In practice, use RST to sharpen the first steps over 0–10 m, where our meta-analysis found the biggest gains, especially in youth. Keep UST on other days to maintain high-speed running, since overall sprint outcomes did not clearly favor RST. In team sports, use RST to help players speed up again after braking and turning; if training already includes sprint and agility work, the extra benefit may be smaller, so pair RST with COD technique and eccentric strength. In court and jump-dominant sports, rely on plyometrics to raise jump height and use light-load RST to boost the first step and approach speed. For youth, start with loads that make runs about 10%–20% slower than unresisted and progress gradually with close technical coaching. For adults or well-trained athletes, target the main limiter in short blocks, such as early-step propulsion or top-speed exposure. RST also outperformed RT controls in our data, but mid-phase sprint gains were less consistent, so combine RST with UST or dedicated speed sessions to build both the start and top-speed ends.

### 5.1 Future direction and limitation

This study has several limitations. Variability in participant characteristics (e.g., sport type, sex, training level) and intervention parameters (e.g., sled load, sprint distance, training frequency, total volume) may have influenced the findings. Although subgroup analyses by age (youth vs. adults) and control type were performed, uncontrolled factors may still confound the results. Moreover, most included studies involved male soccer players, limiting the generalizability of the conclusions to female athletes or those in other sports. Finally, the small number of studies on change-of-direction and vertical jump performance reduces confidence in the pooled estimates. More high-quality randomized controlled trials are needed to confirm the effects of RST on these outcomes and to refine optimal load prescriptions.

Future studies should aim to address these limitations and further optimize the application of RST. First, individualized load prescriptions should be clarified and explored, as no consensus currently exists on the “optimal load” for sprinting, jumping, or change-of-direction performance. Second, longer-term longitudinal trials are needed to evaluate the sustainability of RST effects and to determine whether progressive overload principles can yield additional gains. Third, within a periodized plan, pair RST with squats or jump squats, plyometrics, contrast or complex sets, COD or deceleration drills, and, on some days, UST or AST; early work suggests these combinations can be synergistic (Loturco et al., 2017; Zghal et al., 2019; Pareja-Blanco et al., 2021; Hermosilla-Palma et al., 2025). Finally, beyond competitive performance, future research should also examine the role of RST in injury prevention and potential injury risks, thereby broadening its practical value.

## 6 Conclusion

This meta-analysis systematically examined the effects of RST on athletes’ linear acceleration, vertical jump, and change-of-direction performance, and highlighted its differences from UST. RST and UST both enhance linear sprint performance and COD ability. RST additionally produces a significant within-group improvement in vertical jump and outperforms UST for initial acceleration (0–10 m), with the advantage most evident in youth athletes. In practice, coaches aiming to improve start acceleration and COD should favor RST; to build comprehensive explosive power, combine RST with vertically oriented strength or plyometric work to develop vertical and horizontal movement capacities.

## Data Availability

The original contributions presented in the study are included in the article/Supplementary Material, further inquiries can be directed to the corresponding author.
