# Supplementary material for: Effects of resisted sprint training on sprint, jump, and change-of-direction performance in athletes: a systematic review and meta-analysis
Source: Front Physiol. 2025 Nov 26;16:1711992. doi: 10.3389/fphys.2025.1711992 (PMC12689304; doi:10.3389/fphys.2025.1711992)
Supplement: Supplementary file 1 [file Table1.docx]

| Database | Web of Science (all database) | |
| --- | --- | --- |
| Search Date | July 12, 2025 | |
| Search Period | From the inception of database to July 12, 2025 | |
| No. | Search strategy | Literatures retrieved |
| #1 | TS=(resisted sprint* OR sled train* OR weighted sprint* OR parachute sprint* OR sprint towing) | 3,194 |
| #2 | TS=(jump performance OR vertical jump OR squat jump OR countermovement jump OR drop jump OR reactive strength index OR jump height OR sprint performance OR sprint speed OR sprint time OR linear sprint OR acceleration OR change-of-direction performance OR agility OR COD OR CODS OR "505 test" OR "T-test") | 824,147 |
| #3 | TS=(athlete* OR player* OR "team sport*" OR sportspeople) | 308,617 |
| #4 | #3 AND #2 AND #1 and Preprint Citation Index (Exclude – Database) | 1,469 |

| Database | PubMed | |
| --- | --- | --- |
| Search Date | July 12, 2025 | |
| Search Period | From the inception of database to July 11, 2025 | |
| No. | Search strategy | Literatures retrieved |
| #1 | "resisted sprint"[Title/Abstract] OR (("resist"[All Fields] OR "resistance"[All Fields] OR "resistances"[All Fields] OR "resistant"[All Fields] OR "resistants"[All Fields] OR "resisted"[All Fields] OR "resistence"[All Fields] OR "resistences"[All Fields] OR "resistent"[All Fields] OR "resistibility"[All Fields] OR "resisting"[All Fields] OR "resistive"[All Fields] OR "resistively"[All Fields] OR "resistivities"[All Fields] OR "resistivity"[All Fields] OR "resists"[All Fields]) AND "running"[Title/Abstract]) OR "sled train*"[Title/Abstract] OR "sled tow*"[Title/Abstract] OR "sled pull*"[Title/Abstract] OR "weighted sled"[Title/Abstract] OR (("aviation"[MeSH Terms] OR "aviation"[All Fields] OR "parachuting"[All Fields] OR "parachute"[All Fields] OR "parachuted"[All Fields] OR "parachutes"[All Fields]) AND "sprint*"[Title/Abstract]) OR "weighted vest"[Title/Abstract] OR "tethered sprint"[Title/Abstract] OR (("harness"[All Fields] OR "harnessed"[All Fields] OR "harnesses"[All Fields] OR "harnessing"[All Fields]) AND "sprint"[Title/Abstract]) OR "uphill sprint*"[Title/Abstract] OR (("inclinable"[All Fields] OR "inclination"[All Fields] OR "inclinations"[All Fields] OR "incline"[All Fields] OR "inclined"[All Fields] OR "inclines"[All Fields] OR "inclining"[All Fields]) AND "sprint*"[Title/Abstract]) | 3,447 |
| #2 | "jump"[All Fields] OR "vertical jump"[All Fields] OR "jump height"[All Fields] OR "countermovement jump"[All Fields] OR "CMJ"[All Fields] OR "agility"[All Fields] OR "change-of-direction"[All Fields] OR "change-of-direction"[All Fields] OR "COD"[All Fields] OR "505 test"[All Fields] OR "T-test"[All Fields] OR "sprint performance"[All Fields] OR "sprint speed"[All Fields] OR "acceleration"[All Fields] | 248,251 |
| #3 | "athlete*"[All Fields] OR "player*"[All Fields] OR "team sport*"[All Fields] OR "sportspeople"[All Fields] | 162,837 |
| #4 | #3 AND #2 AND #1 | 286 |
|  |  |  |

| Database | Embase | |
| --- | --- | --- |
| Search Date | July 12, 2025 | |
| Search Period | From the inception of database to July 12, 2025 | |
| No. | Search strategy | Literatures retrieved |
| #1 | 'resistance training'/exp AND 'running'/exp OR 'resisted sprint':ti,ab OR 'resisted sprinting':ti,ab OR 'sled training':ti,ab OR 'sled towing':ti,ab OR 'sled pulling':ti,ab OR 'parachute sprinting':ti,ab OR 'parachute training':ti,ab OR 'weighted vest':ti,ab OR 'tethered sprint':ti,ab OR 'uphill sprint':ti,ab | 1,572 |
| #2 | 'jump':ti,ab OR 'vertical jump':ti,ab OR 'countermovement jump':ti,ab OR 'jump height':ti,ab OR 'drop jump':ti,ab OR 'cmj':ti,ab OR 'sprint performance':ti,ab OR 'sprint speed':ti,ab OR 'acceleration':ti,ab OR 'agility':ti,ab OR 'change of direction':ti,ab OR 'change-of-direction':ti,ab OR 'cod':ti,ab OR '505 test':ti,ab | 142,299 |
| #3 | 'athlete'/exp OR athlete*:ti,ab OR players:ti,ab OR sportsman:ti,ab OR sportspeople:ti,ab OR footballers:ti,ab OR sprinters:ti,ab | 181,855 |
| #4 | #3 AND #2 AND #1 | 350 |

| Database | MEDLINE | |
| --- | --- | --- |
| Search Date | July 12, 2025 | |
| Search Period | From the inception of database to July 12, 2025 | |
| No. | Search strategy | Literatures retrieved |
| #1 | AB ("resisted sprint" OR "sled training" OR "sled towing" OR "sled pulling" OR "parachute sprint" OR "parachute training" OR "weighted sled" OR "weighted vest" OR "tethered sprint" OR "uphill sprint") | 319 |
| #2 | AB( "jump" OR "vertical jump" OR "countermovement jump" OR "drop jump" OR "jump height" OR "CMJ" OR "sprint performance" OR "sprint speed" OR "acceleration" OR "agility" OR "change of direction" OR "change-of-direction" OR "COD" OR "505 test" ) | 146,737 |
| #3 | AB (athlete* OR sportsman OR sportspeople OR footballer* OR players OR trained individuals OR sprinters) | 163,353 |
| #4 | #3 AND #2 AND #1 | 125 |

| Database | Central | |
| --- | --- | --- |
| Search Date | January12, 2025 | |
| Search Period | From the inception of database to July 12, 2025 | |
| No. | Search strategy | Literatures retrieved |
| #1 | "resisted sprint" OR "resisted sprinting" OR "sled training" OR "sled towing" OR "sled pulling" OR "weighted sled" OR "parachute sprint" OR "parachute training" OR "weighted vest" OR "tethered sprint" OR "uphill sprint" in Title Abstract Keyword - (Word variations have been searched) | 210 |
| #2 | "jump" OR "vertical jump" OR "jump height" OR "countermovement jump" OR "drop jump" OR "CMJ" OR "sprint performance" OR "sprint speed" OR "acceleration" OR "agility" OR "change of direction" OR "change-of-direction" OR "COD" OR "505 test" in Title Abstract Keyword - (Word variations have been searched) | 27,000 |
| #3 | athlete* OR sportsman OR sportspeople OR footballer* OR players OR sprinter* in Title Abstract Keyword - (Word variations have been searched) | 14,740 |
| #4 | #3 AND #2 AND #1 | 68 |

| Database | Scopus | |
| --- | --- | --- |
| Search Date | July 12, 2025 | |
| Search Period | From the inception of database to July 12, 2025 | |
| No. | Search strategy | Literatures retrieved |
| #1 | TITLE-ABS-KEY ( "resisted sprint" OR "resisted sprinting" OR "sled training" OR "sled towing" OR "sled pulling" OR "weighted sled" OR "parachute sprint" OR "parachute training" OR "weighted vest" OR "tethered sprint" OR "uphill sprint" ) | 548 |
| #2 | TITLE-ABS-KEY ( "jump" OR "vertical jump" OR "countermovement jump" OR "drop jump" OR "jump height" OR "CMJ" OR "sprint performance" OR "sprint speed" OR "acceleration" OR "agility" OR "change of direction" OR "change-of-direction" OR "COD" OR "505 test" ) | 701,951 |
| #3 | TITLE-ABS-KEY ( athlete* OR sportsman OR sportspeople OR footballers OR players OR trained individuals OR sprinters ) | 88,107 |
| #4 | #3 AND #2 AND #1 | 52 |

| Database | SPORTdiscus | |
| --- | --- | --- |
| Search Date | July 12, 2025 | |
| Search Period | From the inception of database to July 12, 2025 | |
| No. | Search strategy | Literatures retrieved |
| #1 | AB ("resisted sprint" OR "sled training" OR "sled towing" OR "sled pulling" OR "parachute sprint" OR "parachute training" OR "weighted sled" OR "weighted vest" OR "tethered sprint" OR "uphill sprint") | 346 |
| #2 | AB ("jump" OR "vertical jump" OR "countermovement jump" OR "drop jump" OR "jump height" OR "CMJ" OR "sprint performance" OR "sprint speed" OR "acceleration" OR "agility" OR "change of direction" OR "change-of-direction" OR "COD" OR "505 test") | 36,510 |
| #3 | AB (athlete* OR sportsman OR sportspeople OR footballer* OR players OR sprinter* OR trained individual*) | 441,512 |
| #4 | #3 AND #2 AND #1 | 151 |
